# Relationship between alcohol use disorders, cortisol concentrations, and cytokine levels in patients with sepsis

**DOI:** 10.1186/cc9385

**Published:** 2010-12-22

**Authors:** Marjolein de Wit, Gregory K Wiaterek, Nicole D Gray, Keith E Goulet, Al M Best, John N Clore, Lori B Sweeney

**Affiliations:** 1 Division of Pulmonary Disease and Critical Care Medicine, Department of Internal Medicine, Virginia Commonwealth University, 1200 East Broad Street, Richmond, VA 23298-0050, USA; 2 Department of Biostatistics, Virginia Commonwealth University, 730 East Broad Street, Richmond, VA 23298-0032, USA; 3 Division of Endocrinology and Metabolism, Department of Internal Medicine, Virginia Commonwealth University, 1101 E. Marshall Street, Richmond, VA 23298-0111, USA

## Abstract

**Introduction:**

Patients with alcohol use disorders (AUD) are at increased risk of developing sepsis and have higher mortality. AUD are associated with higher cortisol and anti-inflammatory cytokine profile. Higher cortisol increases risk of death in septic patients. The relationship between AUD and cortisol in septic patients is unknown. We aimed to study this relationship and postulated that AUD would be associated with higher cortisol and anti-inflammatory cytokine profile.

**Methods:**

This was a prospective cohort study of 40 medical intensive care unit (ICU) patients admitted with sepsis. Cortisol, anti-inflammatory interleukin (IL) 10, and pro-inflammatory IL1β, IL6, tumor necrosis factor (TNF) α were measured.

**Results:**

Thirteen (32%) out of 40 patients had AUD. AUD patients had higher cortisol by univariate (39 microg/dl versus 24, *P *= 0.04) and multivariable analyses (44 microg/dl versus 23, *P *= 0.004). By univariate analyses, AUD patients had higher IL10 (198 picog/dl versus 47, *P *= 0.02) and IL6 (527 picog/ml versus 156, *P *= 0.048), but similar IL1β and TNFα. By multivariable analyses, AUD patients had higher IL10 (182 picog/dl versus 23, *P *= 0.049) but similar IL1β, IL6, and TNFα. AUD patients had lower IL1β/IL10 (univariate 0.01 versus 0.10, *P *= 0.04; multivariable 0.01 versus 0.03, *P *= 0.04), lower TNFα/IL10 (univariate 0.15 versus 0.52, *P *= 0.03; multivariable 0.11 versus 0.63, *P *= 0.01), but similar IL6/IL10.

**Conclusions:**

AUD are common diagnoses among medical ICU patients with sepsis. Patients with AUD have higher cortisol concentrations and have differences in cytokine expression. Future studies should seek to determine if these differences may explain the higher severity of illness seen in patients with sepsis and AUD.

**Trial registration:**

ClinicalTrials.gov: NCT00615862

## Introduction

Patients with sepsis who have increased cortisol concentrations or poorer response to adrenocorticotropin hormone (ACTH) stimulation have higher mortality than those with normal cortisol and normal response to ACTH [1-3]. Annane *et al. *found that cortisol >34 microg/dl was associated with a 2.4 higher odds of death compared to ≤34 microg/dl [1]. Lipiner-Friedman *et al. *also found that patients who died of sepsis had higher cortisol compared to those who survived (29 microg/dl versus 24 microg/dl); patients who died also had a lower incremental increase in cortisol in response to ACTH administration (6 microg/dl versus 11 microg/dl) [3]. It is unknown if impaired hypothalamic-pituitary-adrenal (HPA) axis is a marker for increased risk of death or the cause of increased mortality [2].

Alcohol use disorders (AUD) are common problems worldwide [4]. In the United States, 7% of the population has AUD, and among hospitalized patients, the rate of AUD is estimated to be 21 to 42% [5-8]. Patients with AUD are predisposed to developing sepsis, are more likely to require mechanical ventilation, and have a higher risk of death [9-12]. A number of studies have demonstrated higher cortisol in surgical patients with AUD, but not all studies support this finding [13-16]. Individuals with AUD who present for elective outpatient detoxification also have higher cortisol compared to individuals without AUD [17,18].

The reasons for the increased sepsis mortality in patients with AUD may partly be explained by the effects of alcohol on cytokine production. Patients with AUD are known to have altered expression of pro-inflammatory cytokines, including interleukin (IL) 6, tumor necrosis factor (TNF) α, and IL1β [14,16,19,20]. Similarly, anti-inflammatory cytokine (IL10) production has been found to be either elevated or decreased in surgical patients with AUD [15,19]. Patients with AUD have been shown to have a decreased ratio of pro-inflammatory to anti-inflammatory cytokines, a finding that has been linked to the development of nosocomial sepsis [14,19].

Recent studies also demonstrate a link between cortisol and immune function [21,22]. Activation of the HPA axis is associated with immunosuppression, while down-regulation of the axis improves immune function [21,22]. We hypothesized that septic patients with AUD compared to those without AUD might have differences in cortisol and cytokine expression.

The relationship between cortisol and co-existing AUD in critically ill patients with sepsis has not been examined. We conducted an observational pilot study to determine if septic patients with AUD would have higher cortisol compared to septic patients without AUD. We also hypothesized that septic patients with AUD would have more depressed immune function as measured by higher anti-inflammatory cytokine IL10 and lower ratio of pro-inflammatory cytokines (that is, IL1β, IL6, and TNFα) to anti-inflammatory cytokine (that is, IL10). The results of this study have been published in abstract format [23].

## Materials and methods

### Inclusion and exclusion criteria

All patients admitted to the medical ICU from the Emergency Department were evaluated for study eligibility if they met criteria for sepsis as established by the American College of Chest Physicians and Society of Critical Care Medicine Consensus Conference [24]. Exclusion criteria were: age <18, pregnancy, prisoners, no cortisol measured within 24 hours after Emergency Department presentation, etomidate administration prior to cortisol measurement, steroid administration prior to measurement of cortisol, and inability to obtain consent. Of note, no patient was diagnosed with alcohol withdrawal during hospitalization; diagnosis of alcohol withdrawal is based on clinical diagnosis and through monitoring for withdrawal using the Clinical Institute Withdrawal Assessment (CIWA-Ar) [25].

The Virginia Commonwealth University Human Investigation Review Committee approved the study (HM11399) and written informed consent was obtained from patients or legally authorized representatives. To protect patients from adverse consequences related to AUD diagnoses, we also obtained a Certificate of Confidentiality from the National Institutes of Health. The study was registered with ClinicalTrials.gov (NCT00615862). The study was conducted in accordance with the ethical standards of the Declaration of Helsinki.

### Definition of AUD

AUD was ascertained by administering a validated questionnaire to patients or legally authorized representatives (in case patients were unable to respond). The Short Michigan Alcohol Screening Test is a 13-item questionnaire that queries about adverse consequences of alcohol consumption and has been successfully used by other ICU investigators [10,26]. Patients who responded affirmatively to ≥3 questions were considered to have AUD (Table 1). Patients were classified into those with AUD and those without AUD.

**Table 1:** Short Michigan Alcohol Screening Test

An affirmative response to three or more questions is consistent with a diagnosis of alcohol dependence.
1. Is your drinking a problem for you? (By problem we mean do you drink more than other people.)
2. Does your wife, husband, a parent, or other near relative ever worry or complain about your drinking?
3. Do you ever feel guilty about your drinking?
4. Do friends or relatives think you have a drinking problem?
5. Are you unable to stop drinking when you want to?
6. Have you ever attended a meeting of Alcoholics Anonymous (AA)?
7. Has your drinking ever created problems between you and your wife, husband, a parent or other near relative?
8. Have you ever gotten into trouble at work because of your drinking?
9. Have you ever neglected your obligations, your family, or your work for two or more days in a row because you were drinking?
10. Have you ever gone to anyone for help about your drinking?
11. Have you ever been in a hospital because of drinking?
12. Have you ever been arrested for drunken driving, driving while intoxicated, or driving under the influence of alcoholic beverages?
13. Have you ever been arrested, even for a few hours, because of other drunken behavior?

### Cortisol concentrations

Cortisol as measured by the Virginia Commonwealth University Medical Center Department of Pathology was recorded. Quantification of cortisol before and one hour after administration of 250 microg of ACTH was completed within the first 24 hours after Emergency Department presentation. Delta cortisol (Δcortisol) was defined as the incremental increase in cortisol in response to ACTH administration. We defined cortisol as the baseline cortisol (prior to ACTH administration). Cortisols were tested in samples within one hour after collection using the ADVIA Centaur cortisol assay (Bayer, Tarrytown, NY, USA), which is a competitive immunoassay using direct chemiluminescent technology.

### Cytokine quantification

Investigators measured IL10, IL1β, IL6, and TNFα on the serum sample from which the baseline cortisol was measured. If insufficient volume of serum remained, cytokines were not measured. Patients did not have blood drawn exclusively for study purposes. Samples were stored at -80 degrees Celsius. Cytokines were measured using Milliplex AP Assay which is based on the Luminex xMAP technology (Millipore Corporation, Billierica, MA, USA) and measures cytokines using antibody techniques.

### Other variables

Demographics, infection site and type, mechanical ventilation characteristics, length of stay, and mortality were also recorded. Severity of illness as measured by Acute Physiology and Chronic Health Evaluation II (APACHE II) and Sequential Organ Failure Assessment (SOFA) were computed [27,28].

### Data analysis

The primary endpoint of the study was the comparison in baseline cortisol between the two groups of patients. Because we expected different demographics between the two groups (that is, demographics such as age, and co-morbidities such as cirrhosis), we also adjusted for between group characteristics when the *P *≤ 0.25. Multivariable analysis using the standard least square was performed and included all possible two-way interactions. We set α = 0.05, and we did not adjust for multiple comparisons in this pilot study.

Secondary outcomes were between group comparison of Δcortisol, cytokines (IL10, IL1β, IL6, and TNFα), and pro-inflammatory to anti-inflammatory cytokine ratios (IL1β/IL10, IL6/IL10, and TNFα/IL10).

A two-sided t-test was used when the outcome variable was continuous after appropriate logarithmic conversion when variables were not normally distributed. Homogeneity of variance was found to be present using the Brown-Forsythe test. Chi Square Test or Fisher's Exact Test was used when the outcome variable was categorical. A log-rank test was used to compute mechanical ventilation duration and ICU and hospital lengths of stay.

Normally distributed data are reported as mean and 95% confidence interval (CI). Non-normally distributed data are reported as median and interquartile range.

### Sample size calculation

Spies *et al. *reported cortisol concentrations in surgical patients with AUD [16]. Based on their data, the AUD group had a mean cortisol one day after surgery of 750 nmol/l. We estimated that the group of patients without AUD had a mean 250 nmol/l. We assumed the data were lognormal since the mean was not symmetric within the interquartile range, and we concluded that a 10% increase in cortisol between the patients without and those with AUD would be clinically relevant. Conservatively, the interquartile range was ± standard deviation. Thus the standard deviation was approximately half the interquartile range. Assuming at least a 10% increase in the mean cortisol in the group of patients with AUD compared to the group without AUD, we determined that a sample size of 40 patients in total would be associated with a 90% power at an α = 0.05. Sample size calculation was performed using nQuery Advisor (version 7.0, Statistical Solutions Ltd., Cork, Ireland).

## Results

Between July 2008, and May 2009, 137 patients were admitted with a diagnosis of sepsis from the Emergency Department. Thirty-four patients did not have cortisol measured, 26 received etomidate, 21 received steroids (either stress dose or were on chronic steroids), 10 were prisoners, and 6 declined study participation.

Forty patients were enrolled, and 13 were diagnosed with AUD (32%). All patients with AUD were actively drinking at the time of hospital admission, and no patient was diagnosed with alcohol withdrawal during their hospitalization (that is, CIWA-Ar <10 in all cases). The demographics of the cohort are detailed in Table 2. Patients with AUD tended to be younger, tended to have lower glucose concentrations, tended to have cirrhosis more frequently, and tended to require vasopressor support more frequently; these four variables were included in multivariable analyses. The lung was the most common site of infection. There was no difference between the two groups in the need for and duration of mechanical ventilation, lengths of stay, and mortality (Table 2).

**Table 2:** Characteristics of the patients with and without alcohol use disorders (AUD)

	AUD Present	AUD Absent	*P*
n	13	27	
Age *	47 (37.1; 56.3)	56 (49.8; 63.2)	0.10
Male (n, %)	7 (54%)	11 (41%)	0.44
Race (African American/white) (n, %)	8 (62%)/5 (38%)	16 (59%)/11 (41%)	0.89
APACHE II *	24 (18.3; 29.2)	22 (18.2; 25.8)	0.59
SOFA *	10 (7.1; 12.2)	8 (6.2; 10.0)	0.28
Vasopressor required within first 24 hours (n, %)	8 (62%)	9 (33%)	0.17
Glucose **	110 (92.5; 128.0)	141 (105.0; 233.0)	0.06
Cirrhosis (n, %)	3 (23%)	2 (7%)	0.18
Hours after admission blood sample collected *	9 (3.5; 13.8)	11 (7.8; 14.9)	0.38
Site of infection (n, %)			0.98
Lung	7 (54%)	11 (41%)	
Blood	2 (15%)	6 (22%)	
Urinary tract	2 (15%)	5 (19%)	
Gastrointestinal	1 (8%)	2 (7%)	
Skin/soft tissue	1 (8%)	2 (7%)	
Gynecologic	0 (0%)	1 (4%)	
Bacteremia (n, %)	5 (38%)	11 (41%)	0.89
Gram positive organisms (n, %)			0.32
Staphylococcus aureus	1 (8%)	5 (19%)	
Enterococcus species	0 (0%)	3 (11%)	
Streptococcus pneumoniae	1 (8%)	1 (4%)	
Other streptococcus species	1 (8%)	1 (4%)	
Other staphylococcus species	0 (0%)	1 (4%)	
Other gram positive organisms	1 (8%)	0 (0%)	
Gram negative organisms (n, %)			0.83
Klebsiella pneumoniae	2 (15%)	3 (11%)	
Pseudomonas species	0 (0%)	3 (11%)	
Eschericia coli	1 (8%)	2 (7%)	
Enterobacter species	0 (0%)	1 (4%)	
Other gram negative organisms	0 (0%)	2 (7%)	
Required mechanical ventilation (n, %)	8 (62%)	16 (59%)	0.89
Mechanical ventilation duration ***	4 (0.8; 5.4)	4 (1.1; 6.1)	0.59
ICU mortality	3 (23%)	5 (19%)	0.74
ICU length of stay **	3 (1.0; 5.6)	5 (2.8; 7.8)	0.33
Hospital mortality	3 (23%)	5 (19%)	0.74
Hospital length of stay **	8 (4.7; 16.0)	9 (6.2; 12.2)	0.82

* mean, 95% confidence interval.

** median, interquartile range.

AUD, alcohol use disorders; APACHE II, Acute Physiology and Chronic Health Evaluation II; SOFA, Sequential Organ Failure Assessment; ICU, Intensive Care Unit.

By univariate analysis, patients with AUD had significantly higher cortisol levels (Table 3). Multivariable analysis also demonstrated AUD was an independent predictor of higher cortisol.

**Table 3:** Univariate and multivariable analyses of cortisol and cytokine concentrations in the two groups of patients

	Univariate analysis			Multivariable analysis**		
		
	AUD present	AUD absent	p	AUD present	AUD absent	*P*
Cortisol (microg/dl) *	39 (27.0; 60.0)	24 (18.7; 31.4)	0.04	44 (31.0; 62.9)	23 (8.0; 29.1)	0.004
IL10 (picog/ml) *	198 (63.1; 621.5)	47 (20.1; 110.7)	0.02	182 (51.0; 646.4)	23 (6.8; 80.0)	0.049
IL6 (picog/ml) *	527 (154.6; 1794.2)	156 (72.0; 339.2)	0.048	641 (169.1; 2426.5)	137 (40.4; 465.1)	0.10
TNFα (picog/ml) *	30 (18.3; 50.4)	24 (17.0; 34.8)	0.23	44 (25.9; 74.9)	25 (15.9; 40.4)	0.06
IL1β (picog/ml) *	2 (0.4; 14.9)	6 (1.5; 21.1)	0.41	8 (1.1; 56.5)	3 (0.7; 14.7)	0.82
IL6/IL10 *	3.0 (1.27; 6.93)	4.9 (2.80; 8.67)	0.17	3 (1.2; 8.0)	8 (3.3; 19.5)	0.32
TNFα/IL10 *	0.15 (0.005; 0.437)	0.52 (0.240; 1.139)	0.03	0.11 (0.041; 0.292)	0.63 (0.307; 1.293)	0.01
IL1β/IL10	0.01 (0.001; 0.052)	0.10 (0.020; 0.530)	0.04	0.01 (0.001; 0.029)	0.03 (0.07; 0.104)	0.04

* mean, 95% confidence interval.

** Adjusted for age, glucose, cirrhosis, and vasopressor use.

AUD, alcohol use disorders; IL, interleukin; TNF, tumor necrosis factor.

A total of 28 patients underwent quantification of cytokines, 10 with AUD and 18 without AUD. By univariate analyses, patients with AUD had higher IL10 and IL6, but lower IL1β/IL10 and TNFα/IL10 (Table 3). Multivariable analyses revealed that patients with AUD had higher IL10, but lower IL1β/IL10 and TNFα/IL10.

The study was started after publication of the CORTICUS trial, and only 15 patients underwent administration of ACTH, 5 in the group with AUD and 10 in the group without AUD [29]. Because of this small number of patients, the data are not reported in table format. By univariate analysis, patients with AUD had a smaller Δcortisol compared to the patients without AUD: 6 microg/dl, 95% CI (3.1; 10.5) versus 12 microg/dl, 95% CI (8.0; 19.0), *P *= 0.04. Multivariable analysis demonstrated that AUD was an independent predictor for lower Δcortisol: Patients with AUD had a Δcortisol of 3 microg/dl, 95% CI (1.3; 6.0) versus 7 microg/dl, 95% CI (3.8; 12.4), *P *= 0.01. When using a Δcortisol ≤9 microg/dl as a diagnostic cutoff of relative adrenal insufficiency, the two groups had similar rates of relative adrenal insufficiency by univariate analysis (4 out 5 patients with AUD versus 3 out of 10 patients without AUD, *P *= 0.06) and multivariable analysis (*P *= 0.08).

## Discussion

In this prospective observational pilot study, we found that a high proportion of patients with community acquired sepsis have AUD (32%), and that co-diagnoses of AUD are associated with higher cortisol concentrations. In secondary analyses, we found that patients with and without AUD had differences in cytokine expression. Patients with AUD had higher levels of the anti-inflammatory cytokine IL10 but there was no difference in pro-inflammatory cytokines IL1β, IL6, and TNFα. Patients with AUD had an anti-inflammatory cytokine profile, as measured by depressed ratios of IL1β/IL10 and TNFα/IL10; however, the ratio of IL6/IL10 was similar.

AUD have been associated with HPA dysfunction in ambulatory individuals [17,18,30]. A majority of the literature finds that surgical ICU patients with AUD have higher cortisol concentrations [13-16]. In our current study, we similarly determined that septic patients with co-diagnoses of AUD had higher cortisol: AUD was associated with a 1.9-fold higher concentration by multivariable analysis. We do not believe the increased cortisol concentrations in patients with AUD were caused by the development of alcohol withdrawal syndrome as no patient was diagnosed with this complication during hospitalization (as measured every four hours by CIWA-Ar which is our standard of care).

Patients with AUD had similar pro-inflammatory cytokines concentrations (IL1β, IL6, and TNFα) but had higher levels of anti-inflammatory cytokine IL10 compared to patients without AUD (Table 3). These findings are supported by other studies. Anti-inflammatory IL10 is elevated in the immediate post-operative period in patients with AUD [15,19]. Studies examining surgical patients have found conflicting results on the levels of pro-inflammatory cytokine TNFα and IL1β [14]. IL6 levels in patients with AUD have been found to be similar, higher, or lower than patients without AUD [14,15,19]. The increased IL10 concentration in patients with AUD resulted in a lower ratio of IL1β/IL10 and TNFα/IL10 but a similar ratio of IL6/IL10, findings supported by other studies [14,19]. The differences observed between our study results and other studies may be explained by the timing of cytokine measurement (within 24 hours of admission) and the patient population studied. Other studies have examined post-operative patients while our study evaluated medical ICU patients admitted with sepsis.

The implications of this pilot study are that septic medical ICU patients with AUD exhibit heightened stress response and less robust immune response in the setting of life threatening sepsis.

Our study has several limitations. Because we started our study after publication of the CORTICUS study, only 15 out of 40 patients underwent stimulation with ACTH, and the small number of patients in whom Δcortisol could be computed limits generalizability [3]. We also did not find a difference in mortality between patients with and without AUD, but we did not power our study to detect this difference. A larger study powered to detect mortality differences needs to be conducted. In addition, we found an association between AUD and cortisol and immune function, but the observational nature of our study does not permit determination of cause and effect. Cortisol and cytokine levels were determined on one occasion and were not evaluated longitudinally over time. In addition, we measured cytokine concentrations only if sufficient serum volume remained, leading to potential bias of test results: Two-thirds of patients without AUD had cytokines measured while three-quarter of patients with AUD had cytokines quantified. In the acute phase of sepsis, cytokines change over time. Our measurement at one point in time does not fully reflect the interactions between AUD and systemic inflammation. In this exploratory study, we also did not adjust α for multiple analyses, and future studies enrolling with adequate power and enrolling larger number of patients need to be conducted. Finally, only 29% of septic patients were enrolled in the study, and we do not have demographics on non-enrolled patients. It is possible that the enrolled patients were qualitatively different from those not enrolled, and that these difference may have impacted our results.

## Conclusions

In conclusion, AUD are common co-diagnoses among patients with sepsis, affecting approximately one-third of patients. AUD are associated with higher cortisol concentrations and a different cytokine composition. Anti-inflammatory cytokine IL10 is increased and the ratios of IL1β/IL10 and TNFα/IL10 are lower in patients with AUD, suggesting that AUD may be associated with immunosuppression. Future studies should aim to determine if these differences may be the cause of higher morbidity and mortality experienced by patients with AUD.

## Key messages

• In septic medical ICU patients, patients with co-existing AUD have higher cortisol concentrations compared to patients without AUD.

• Septic patients with AUD have differences in cytokine composition compared to septic patients without AUD.

## Abbreviations

ACTH: adrenocorticotropin hormone; AUD: alcohol use disorders; CI: confidence interval; CIWA-Ar: Clinical Institute Withdrawal Assessment; Δcortisol: delta cortisol; HPA: hypothalamic-pituitary-adrenal; ICU: intensive care unit; IL: interleukin; TNF: tumor necrosis factor.

## Competing interests

The authors declare that they have no competing interests.

## Authors' contributions

MdW participated in study design, data collection, data analysis and interpretation, and manuscript preparation. GKW, NDG and KEG participated in data collection, data analysis, and manuscript preparation. LBS and JNC participated in study design, data analysis and manuscript preparation.
